# Case Report: A somatic *NF1* splice-altering variant identified in lesional tissue in peripheral blood-negative segmental facial neurofibromatosis

**DOI:** 10.3389/fnins.2026.1887523

**Published:** 2026-08-12

**Authors:** Song Su, Haochen Zou, Yinhua Lin, Huafang Jiang, Ying Ren, Wenchao Zhang, Wandong Hu, Deyue Li, Wei Zhang, Hongwei Zhang

**Affiliations:** 1 Neurology Department, Children's Hospital Affiliated to Shandong University, Jinan, Shandong, China; 2 Neurology Department, Jinan Children’s Hospital, Jinan, Shandong, China; 3 Pediatrics, Yanbian University Medical College, Yanji, Jilin, China; 4 Pediatrics, Anqiu People's Hospital, Weifang, Shandong, China; 5 Neurology Department, WeiFang Maternal and Child Health Hospital, Weifang, Shandong, China; 6 Emergency department, Children’s Hospital Affiliated to Shandong University, Jinan, Shandong, China

**Keywords:** immunohistochemical, minigene assay, neurofibromin, NF1, segmental neurofibromatosis, somatic mosaicism, splicing mutation

## Abstract

**Background:**

Neurofibromatosis type 1 (NF1) is a common autosomal dominant disorder caused by loss-of-function variants in the *NF1* gene. Segmental neurofibromatosis represents a rare mosaic form resulting from postzygotic mutations and is often diagnostically challenging due to its localized presentation and variable clinical expressivity.

**Methods:**

A 25-year-old woman with neurofibromas restricted to the left facial region underwent histopathological evaluation and whole exome sequencing (WES) of lesional tissue. Bioinformatic analyses were used to predict the splicing impact of a novel intronic variant. A minigene splicing assay was performed to experimentally assess transcript-level consequences. Protein structural modeling was used to evaluate the predicted effects on neurofibromin.

**Results:**

We describe a 25-year-old woman with a segmental distribution of neurofibromas confined to the left facial region, with a progressive mass present for more than two decades. Histopathological and immunohistochemical evaluation of the lesional tissue was performed, followed by WES of paired peripheral blood and affected tissue. WES of the affected tissue identified a novel somatic intronic deletion, *NF1* c.7970+4_7970+7del, with a mosaic variant allele fraction of 36.02%. In silico analyses predicted disruption of the canonical donor splice site. A minigene splicing assay experimentally confirmed that the variant causes complete exon 54 skipping, which is predicted to result in a frameshift and premature termination. These findings provide functional evidence that NF1 c.7970+4_7970+7del is a splice-disrupting variant with pathogenic relevance in the context of segmental neurofibromatosis.

**Conclusion:**

This case expands the spectrum of splice-altering *NF1* variants and reveals a previously uncharacterized molecular mechanism underlying segmental neurofibromatosis. The mosaic nature of this somatic variant has important genetic counseling implications, including the possibility of low-level germline involvement. Our results highlight the value of functional validation for intronic variants of uncertain significance and support the potential role of early molecular diagnosis in guiding targeted therapeutic approaches such as MEK inhibition.

## 1 Introduction

Neurofibromatosis type 1 (*NF1*) is a common autosomal dominant neurocutaneous disorder caused by pathogenic variants in the *NF1* gene on chromosome 17q11.2. Affecting approximately 1 in 3,000 individuals, NF1 presents with a broad phenotypic spectrum that includes café-au-lait macules, axillary or inguinal freckling, Lisch nodules, osseous dysplasia, and benign or malignant tumors of the peripheral and central nervous systems (Rasmussen and Friedman, 2000). The clinical variability of NF1 reflects both the complexity of neurofibromin function and the extensive mutational heterogeneity of the *NF1* gene. Neurofibromin is a large 2818-amino-acid cytoplasmic protein that functions as a RAS GTPase-activating protein (RAS-GAP), thereby serving as a central negative regulator of the RAS/MAPK signaling cascade. Loss of neurofibromin activity leads to uncontrolled RAS activation, increased cellular proliferation, and predisposition to benign and malignant neoplasms (Heim et al., 1994).

To date, more than 3,000 pathogenic *NF1* variants have been identified, including nonsense, frameshift, missense, splice-site, and large genomic rearrangements. Among these, splice-altering variants represent a particularly challenging subgroup. Approximately 10–15% of pathogenic *NF1* variants affect RNA splicing, but this proportion is likely underestimated because many intronic and exonic variants outside canonical splice sites are difficult to interpret using DNA-based testing alone (Bergoug et al., 2020; Cichowski and Jacks, 2001; Fahsold et al., 2000; Griffiths et al., 2007; Mcclatchey, 2007; Messiaen et al., 2000; Scheffzek and Welti, 2012; Upadhyaya et al., 2008; Van Minkelen et al., 2014). Noncanonical splice-site variants—such as those in the +3 to +7 donor region or −3 to −20 acceptor region may significantly disrupt pre-mRNA processing while appearing benign at the DNA level. Consequently, RNA analysis or minigene splicing assays have become essential tools for establishing pathogenicity, especially when variants reside in intronic regions not routinely assessed by clinical RNA testing.

Segmental neurofibromatosis, also referred to as mosaic NF1, is a rare subtype characterized by pigmentary changes or neurofibromas confined to a circumscribed body region (Ruggieri and Huson, 2001; Sobjanek et al., 2014; Tinschert et al., 2000). This phenotype results from postzygotic somatic mutations affecting a subset of embryonic precursor cells, producing a mixture of mutant and wild-type cell lineages. The extent of clinical involvement depends on the developmental timing of the mutation: mutations arising earlier in embryogenesis produce wider tissue distribution and occasionally systemic features, whereas later events produce sharply demarcated, localized disease (Consoli et al., 2005; Sobjanek et al., 2014). Despite its distinctive clinical presentation, the molecular basis of segmental *NF1* remains poorly understood, and few cases have been characterized at the genomic and functional level. Moreover, mosaic NF1 poses specific challenges in diagnosis, recurrence-risk estimation, and therapeutic decision-making, particularly because low-level mosaicism may escape detection in blood while being present at higher levels in affected tissues.

In this study, we describe a patient with segmental neurofibromatosis limited to the left facial region, in whom whole exome sequencing (WES) of lesional tissue identified a novel somatic intronic deletion, *NF1* c.7970+4_7970+7del. This variant lies within the donor splice-site region of intron 54, a position known to influence U1 snRNP recognition and spliceosome assembly. Predictive algorithms suggested that the deletion disrupts normal splicing; however, its molecular impact could not be definitively assessed without functional validation. Using an *in vitro* minigene assay, we demonstrate that this intronic deletion abolishes normal splicing and causes complete skipping of exon 54, generating a frameshift and premature termination codon which may lead to nonsense-mediated mRNA decay or production of a truncated neurofibromin product if translated. Our findings (i) expand the known spectrum of *NF1* splice-altering mutations, (ii) provide mechanistic insight into the molecular basis of segmental *NF1*, and (iii) underscore the importance of functional assays and mosaicism-aware diagnostic approaches for accurate variant classification and genetic counseling.

## 2 Patient information

### 2.1 Essential information

The patient was a 25-year-old woman who presented with a giant facial mass that had persisted for more than two decades. The left-sided facial lesion had progressively enlarged, causing significant cosmetic disfigurement and functional impairment, including restricted mouth opening and compromised mastication. There were no signs of inflammation including redness, swelling, warmth, pain, or tenderness on palpation. Her medical history was unremarkable, with no family history of similar conditions. Physical examination revealed normal growth and development, absence of café-au-lait spots, subcutaneous nodules, iris hamartomas (Lisch nodules) on fundoscopy, skeletal deformities, axillary freckling, or epileptic seizures. Cardiac, respiratory, abdominal, and neurological evaluations showed no abnormalities. Surgical excision of the mass resulted in clinical improvement. Histopathological examination confirmed neurofibromatosis. WES of FFPE lesional tissue identified an *NF1* splice-region variant, c.7970+4_7970+7del, which was initially classified as a variant of uncertain clinical significance. The variant was detected in lesional tissue with a mosaic variant allele fraction, whereas targeted Sanger sequencing of peripheral blood DNA did not detect this variant. Based on the localized distribution of neurofibromas, pathological findings, mosaic NF1 variant in lesional tissue, absence of the variant in peripheral blood by Sanger sequencing, and subsequent functional splicing evidence, the diagnosis of segmental neurofibromatosis was supported.

### 2.2 Auxiliary examination

#### 2.2.1 Routine examination and metabolic analysis

Upon admission, a series of laboratory tests were performed for the patient. The erythrocyte sedimentation rate, white blood cell count, and C-reactive protein (CRP) levels were all within normal limits. In conjunction with the absence of fever and other symptoms, these results suggested that the patient was not experiencing an active infection. Additionally, tests for aspartate aminotransferase, alanine aminotransferase, creatine kinase, creatine kinase isoenzyme, lactate dehydrogenase, urea, creatinine, glucose, and bilirubin all returned normal results, indicating proper functioning of the liver, kidneys, and heart. Blood ammonia, lactic acid, homocysteine, as well as sodium, calcium, potassium, and chloride levels were also within the normal range, ruling out any electrolyte imbalances.

#### 2.2.2 MRI

**Figure 1:**
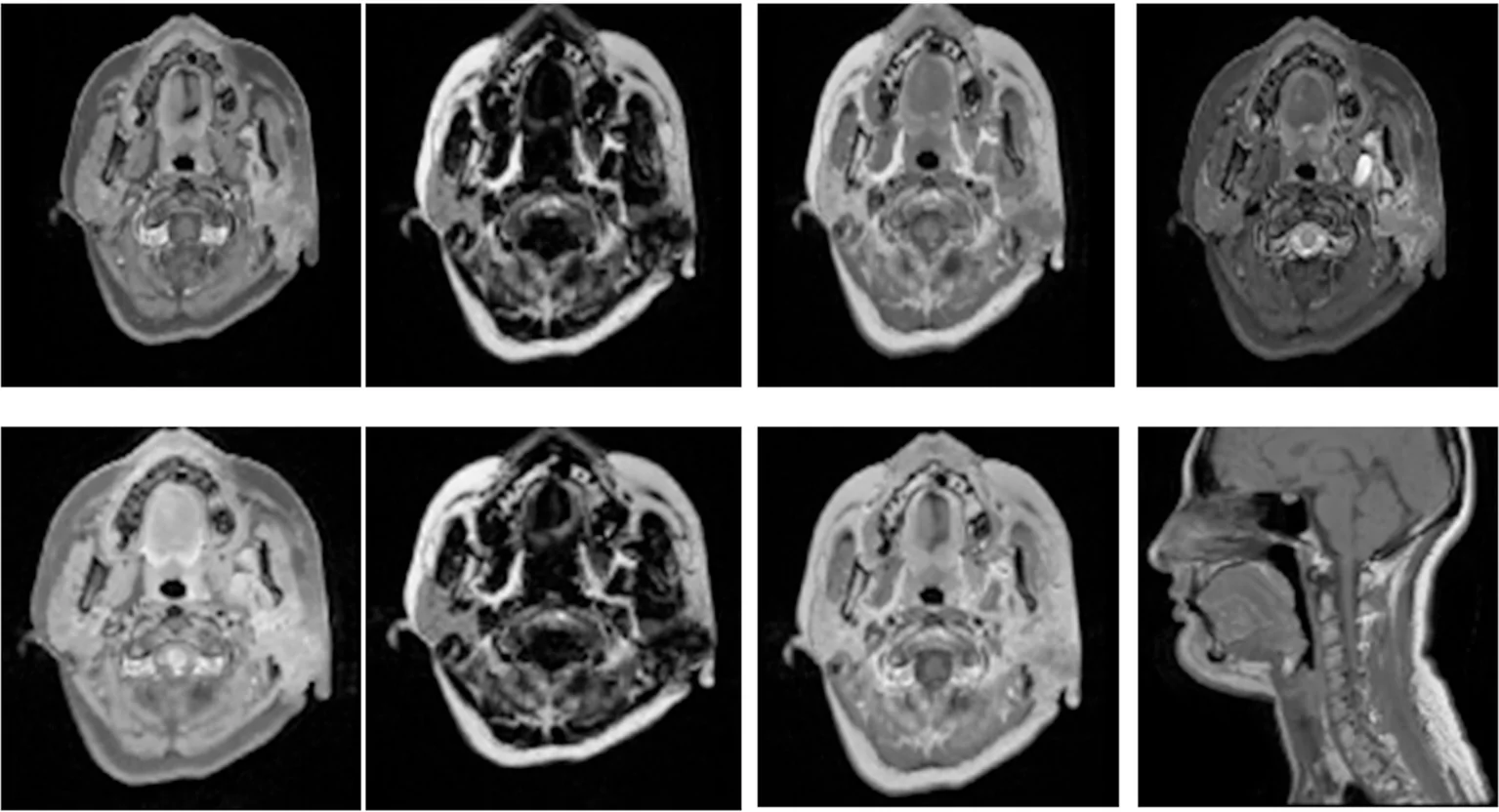
MRI demonstrating a left-sided infiltrative facial neurofibroma. Axial and sagittal MRI sequences show a heterogeneous soft-tissue mass involving the left parotid and adjacent facial spaces, with features consistent with neurofibroma.

Following admission, the patient underwent MRI for further evaluation of the mass. The imaging demonstrated soft tissue thickening with obliterated fat planes in the left parotid region, maxillofacial area, infratemporal fossa, submandibular region, and parapharyngeal space. The lesions exhibited heterogeneous long T1 and T2 signals, appearing hyperintense on fat-suppressed sequences with slightly restricted diffusion and heterogeneous enhancement post-contrast. Serpiginous tubular structures displaying long T1/T2 signals were noted in the left parapharyngeal space, accompanied by adjacent skin thickening. Mildly enlarged lymph nodes were observed in the bilateral cervical and submental regions. The pharyngeal recesses remained symmetric, with preserved epiglottic morphology and no thickening of the bilateral aryepiglottic folds. The imaging findings were consistent with neurofibroma involving the left parotid region, maxillofacial area, infratemporal fossa, submandibular region, and parapharyngeal space, alongside reactive lymphadenopathy in the cervical and submental regions (Figure 1).

#### 2.2.3 Immunohistochemistry of tumor tissues

**Figure 2:**
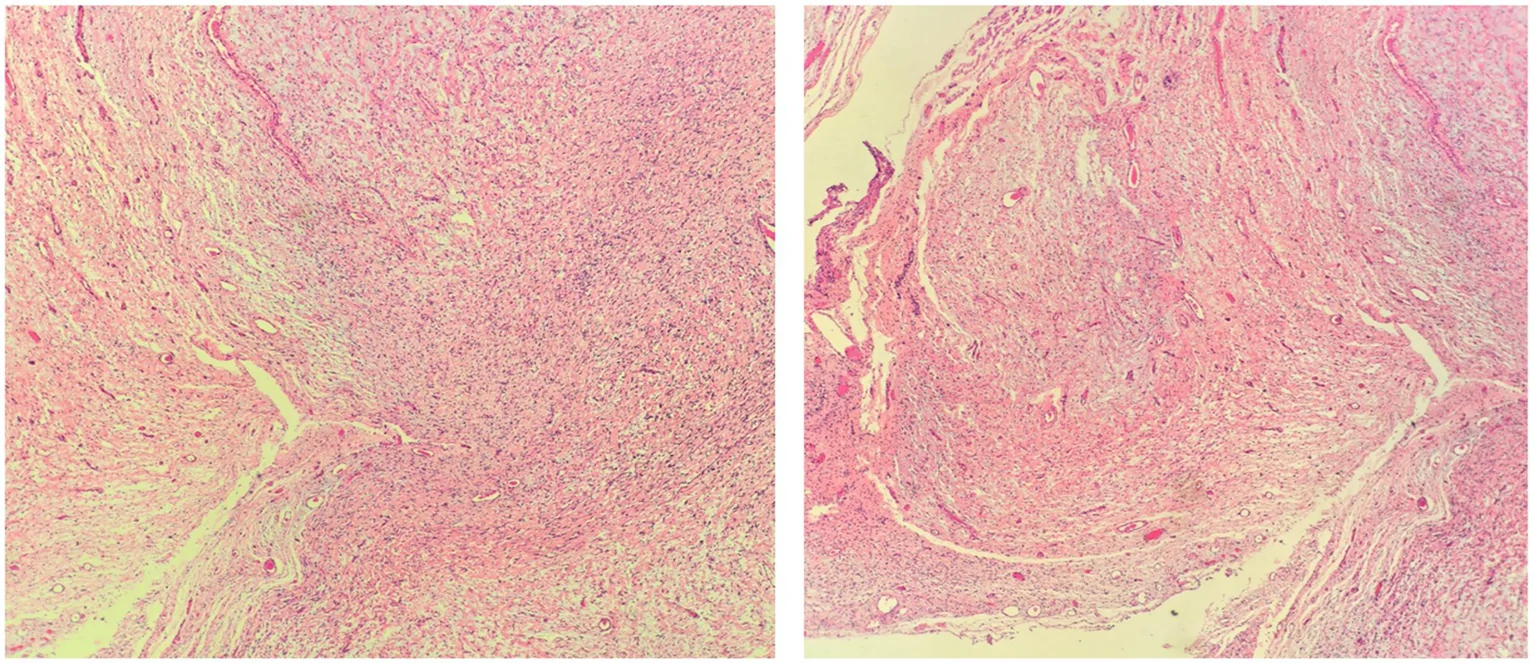
Histopathological features of the facial neurofibroma. HE-stained sections show a poorly circumscribed spindle-cell lesion infiltrating the surrounding soft tissue. The tumor is composed of elongated spindle cells with wavy nuclei embedded in a collagenous and partly myxoid stroma, without significant atypia or mitotic activity. These features are characteristic of benign neurofibroma.

Multiple pieces of facial mass tissue with overlying skin were resected, measuring 5.5 cm × 4 cm × 1.5 cm in aggregate. Gross examination revealed gray-white and gray-yellow cut surfaces with moderate texture, and slightly thickened skin. Microscopically, the facial mass exhibited a spindle cell tumor with ill-defined borders infiltrating surrounding tissues (Figure 2). Immunohistochemical results were as follows: Vimentin (+), SMA (−), Desmin (−), NF (−), S-100 (+), Beta-catenin (−), SOX10 (+), EMA (−), CD34 (+), and Ki-67 (index 2%). These findings are consistent with a diagnosis of neurofibroma.

### 2.3 Treatment

The patient underwent surgical excision of the facial neurofibroma. Although resection improved mass-related functional impairment, residual lesion persisted. Given the involvement of the RAS-MAPK pathway, MEK inhibitor therapy, such as selumetinib, was discussed as a potential therapeutic option. However, this treatment had not been initiated because of financial constraints (Dombi et al., 2016).

## 3 Materials and methods

### 3.1 Compliance with ethical standards

This study was approved by the Medical Ethics Committee of the Children’s Hospital affiliated with Shandong University. Written informed consent was obtained from the parents or legal guardians of the probands prior to conducting any clinical and laboratory assessments. All procedures carried out in this study adhered strictly to the principles outlined in the Declaration of Helsinki.

### 3.2 Whole-exome sequencing

Genomic DNA was extracted from formalin-fixed paraffin-embedded (FFPE) lesional tissue and peripheral blood samples from the proband. For FFPE samples, adjacent sections were reviewed by hematoxylin and eosin (H&E) staining and immunohistochemistry to confirm the presence of lesional cells before DNA extraction. WES including mitochondrial genome analysis, was performed on the FFPE lesional tissue sample. The assay targeted coding exons and adjacent splice-site regions, as well as the full mitochondrial genome. Sequencing reads were aligned to the human reference genome GRCh38/hg38 using BWA. Single-nucleotide variants and small insertions/deletions were called using GATK HaplotypeCaller, followed by annotation and filtering using public population databases, disease databases, in silico prediction tools, and an in-house database. Copy-number variants within the probe-covered regions were analyzed using xhmm and clamms.

Candidate variants were prioritized according to the patient’s clinical phenotype and the relevance of the affected gene to neurocutaneous or tumor-related disorders. Population frequency was assessed using gnomAD and other population databases. Disease relevance was evaluated using databases including OMIM, HGMD, ClinVar, DECIPHER, DGV, and ClinGen. In silico prediction was performed using tools including SIFT, PolyPhen-2, LRT, MutationTaster, FATHMM, M-CAP, CADD, REVEL, dbscSNV, and SpliceAI.

The detected NF1 variant was interpreted according to the 2017 AMP/ASCO/CAP guidelines for sequence variant interpretation in cancer and was initially classified as a Tier III variant of uncertain clinical significance. Its potential clinical and functional relevance was further evaluated based on the patient’s localized phenotype, the mosaic variant allele fraction in lesional tissue, in silico splicing prediction, and experimental minigene splicing analysis.

### 3.3 Sanger sequencing validation

The potentially pathogenic variants detected through WES in the probands were confirmed using Sanger sequencing with specifically designed primers. To evaluate whether the variant was detectable in blood-derived DNA, targeted Sanger sequencing was also performed using peripheral blood DNA from the patient. The primer sets for Sanger validation were created using Primer Premier v5.0 software. Amplification of the target regions was conducted using AmpliTaq Gold 360 DNA polymerase (Applied Biosystems). Subsequently, the PCR products were purified and subjected to sequencing on an ABI Prism 3700 automated sequencer (Applied Biosystems, Foster City, CA, USA).

### 3.4 Bioinformatics analysis of splicing and protein structure

The potential effect of the *NF1* c.7970+4_7970+7del variant on pre-mRNA splicing was evaluated using in silico splicing prediction tools, including SpliceAI and RNA Splicer.^1^ Structural modeling of the wild-type neurofibromin fragment and the putative mutant protei product was performed using SWISSMODEL online tool.^2^

### 3.5 Minigene splicing assay

To evaluate the effect of the NF1 c.7970+4_7970+7del variant on pre-mRNA splicing, an *in vitro* minigene splicing assay was performed. A genomic fragment encompassing NF1 exon 53 to exon 55 was amplified from control genomic DNA and cloned into the pMini-CopGFP vector (Beijing Hitrobio Biotechnology Co., Ltd., Beijing, China) using the ClonExpress II One Step Cloning Kit (Vazyme, Nanjing, China). The mutant construct carrying the c.7970+4_7970+7del variant was generated by site-directed mutagenesis. Both wild-type and mutant constructs were confirmed by Sanger sequencing.

Wild-type and mutant plasmids were transiently transfected into HEK293T cells using Lipofectamine 2000 (Invitrogen, USA). After 48 h, total RNA was extracted using TRIzol reagent (Invitrogen, USA), followed by reverse transcription PCR (RT-PCR) with vector-specific primers. The PCR products were analyzed by agarose gel electrophoresis, and the corresponding splicing isoforms were characterized by Sanger sequencing.

## 4 Results

### 4.1 Genetic analysis

**Figure 3:**
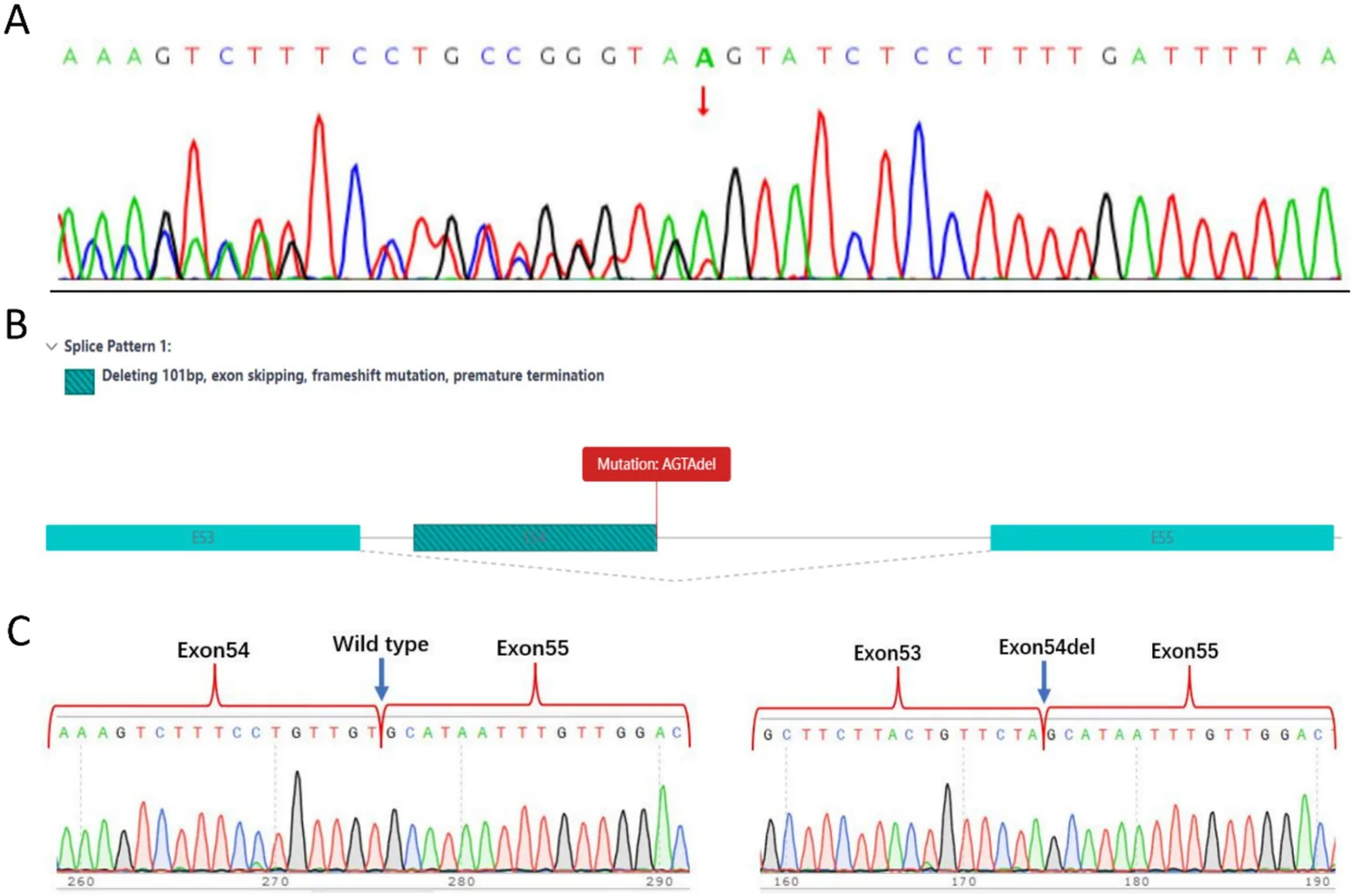
**(A)** Sanger sequencing of tumor DNA reveals the 4-bp deletion c.7970+4_7970+7del (red arrow) at the donor splice site of intron 54 in NF1, producing a clear mixed-peak pattern downstream consistent with a frameshift. **(B)** 3D structure of wild type and mutant type of c.7970+4_7970+7del in NF1, and the mutation of c.7970+4_7970+7del changes the structure. **(C)** Splicing study of c.7970+4_7970+7del by Sanger sequencing of NF1 cDNA clones.

WES of FFPE lesional tissue identified a mosaic intronic deletion in *NF1* gene, NC_000017.11 (NM_001042492.3): c.7970+4_7970+7del (Figure 3A). This variant represents a 4-bp deletion involving the +4 to +7 positions of the donor splice site region immediately downstream of *NF1* exon 54. The deletion was absent from major population databases including gnomAD (East Asian subset), 1,000 Genomes, and ExAC, supporting its rarity and potential clinical significance.

The variant was detected in lesional tissue with a variant allele fraction of 36.02% (wild-type reads: mutant reads = 206:116). Sanger sequencing confirmed the presence of the variant in lesional tissue. To assess whether this variant was detectable in blood-derived DNA, targeted Sanger sequencing was additionally performed using peripheral blood DNA from the patient. The NF1 c.7970+4_7970+7del variant was not detected in peripheral blood, supporting a somatic mosaic origin.

No additional clinically relevant Tier I or Tier II copy-number variants were reported in the WES analysis. No clinically relevant mitochondrial genome variants or ACMG secondary findings were identified. Apart from NF1 c.7970+4_7970+7del, no other reportable variant clearly related to the patient’s phenotype was identified under the laboratory’s reporting criteria.

### 4.2 Bioinformatics analysis

The *NF1* c.7970+4_7970+7del variant is located near the exon-intron boundary and is classified as a splice-altering variant. To evaluate its potential impact on RNA splicing, SpliceAI and web-based RNA splicing prediction tools were utilized (Figure 3B). Computational analysis demonstrated that this variant induces exon 54 skipping. The predicted aberrant transcript was expected to cause a frameshift mutation and the introduction of a premature termination codon.

### 4.3 Splicing study of NF1 c.7970+4_7970+7del by RT-PCR sanger sequencing of the proband

To generate mutant plasmids, site-directed mutagenesis was performed using the primers NF1-MT-F (5′- CCTGTTGTGTATCTCCTTTTGATTTTAATTCACCTTC-3′) and NF1-MT-R (5′- AAGGAGATACACAACAGGAAAGACTTTGGGAAA-3′), followed by validation via Sanger sequencing. The splicing result caused by NF1 c.7970+4_7970+7del was confirmed by RT-PCR Sanger. The results revealed the delete complete exon 54 and translation in the NF1 cDNA with the c.7970+4_7970+7del variant, leading to consequent frameshift mutation with premature termination of protein (Figure 3C).

### 4.4 Splicing study of NF1 c.996-4A>G by minigene assay

**Figure 4:**
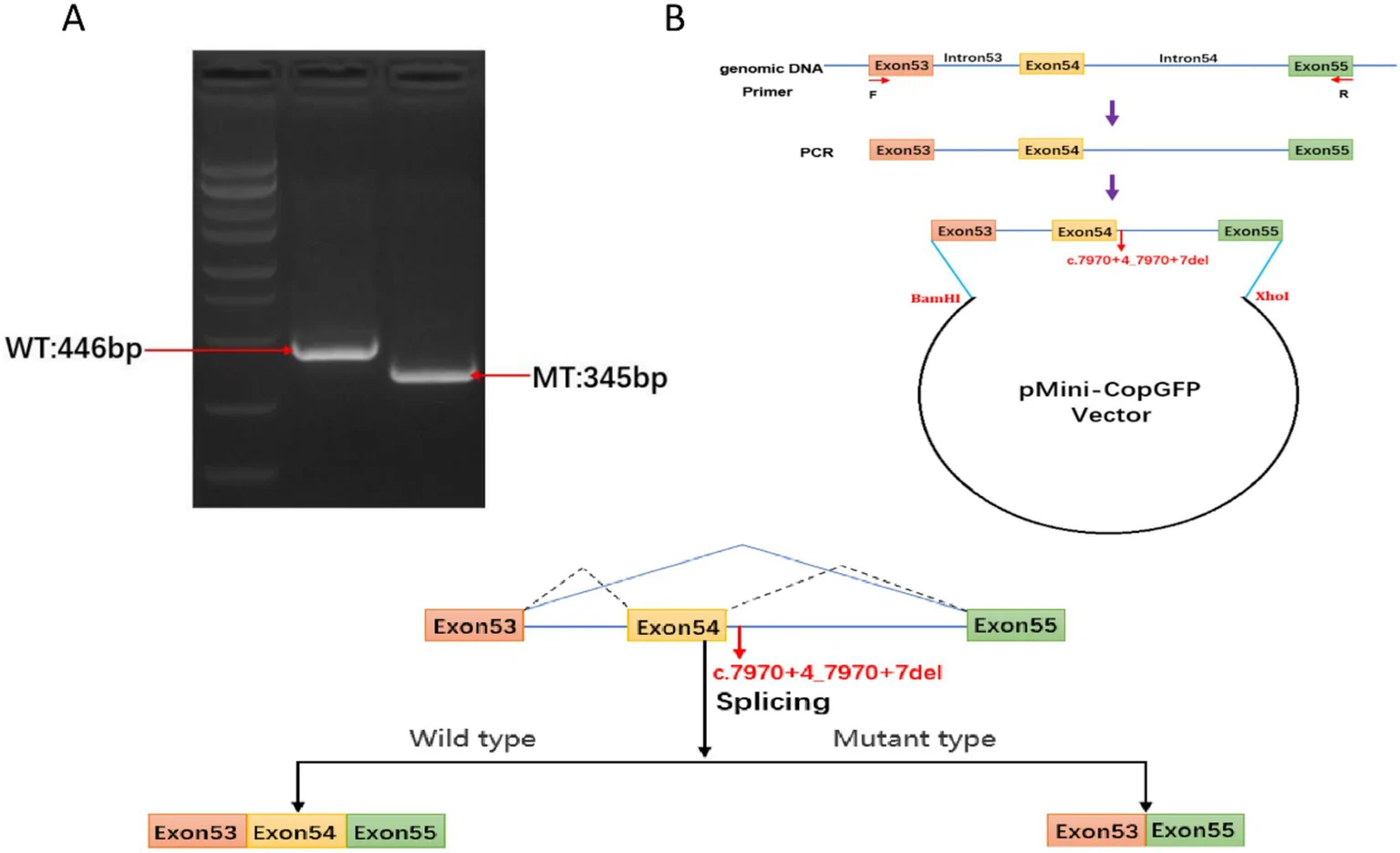
Splicing study of c.7970+4_7970+7del by minigene assay. **(A)** Agarose gel electrophoresis results of RT-PCR for plasmid expression. The wild-type fragment is estimated to be 446 bp, and the mutant type is estimated to 345 bp. WT = wild-type; MT = mutant type. Sanger sequencing of RT-PCR for plasmid expression. WT = wild-type; MT = mutant type. **(B)** Schematic of splicing for c.7970+4_7970+7del in the minigene assay.

To further characterize aberrant splicing caused by the NF1 c.7970+4_7970+7del variant, minigene assays were performed for both wild-type and mutant constructs. Agarose gel electrophoresis of RT-PCR products revealed a single band for the wild-type (expected size: 446 bp) and mutant (expected size: 345 bp), as shown in Figure 4A. Sanger sequencing confirmed that the wild-type produced a normally spliced isoform, whereas the mutant exhibited an aberrant splicing pattern consistent with predictions from the online RNA Splicer tool (Figure 3A). The minigene analysis demonstrated that the c.7970+4_7970+7del variant caused complete skipping of exon 54 in the *NF1* gene, resulting in a frameshift and premature translational termination (p.Thr2625Ter), thereby producing a truncated protein (Figure 4B).

The NF1 c.7970+4_7970+7del variant was initially reported as a Tier III variant of uncertain clinical significance according to the AMP/ASCO/CAP framework for sequence variant interpretation in cancer. The variant was detected in FFPE lesional tissue by whole-exome sequencing, with a mosaic variant allele fraction of 36.02%, and was confirmed by Sanger sequencing. Targeted Sanger sequencing of peripheral blood DNA did not detect this variant, supporting a somatic mosaic origin. No additional clinically relevant Tier I or Tier II copy-number variants, mitochondrial genome variants, or ACMG secondary findings were reported in the WES analysis.

Although the variant was initially classified as a variant of uncertain clinical significance, the minigene splicing assay demonstrated a deleterious effect on NF1 pre-mRNA splicing, resulting in complete exon 54 skipping. This aberrant transcript is predicted to cause a frameshift and premature termination, supporting NF1 loss of function. Together with the absence of the variant from population databases, its mosaic variant allele fraction in lesional tissue, negative peripheral blood Sanger result, and the patient’s segmental distribution of neurofibromas, these findings support the pathogenic relevance of this splice-altering NF1 variant. The localized clinical presentation, mosaic variant allele fraction in lesional tissue, and absence of the variant in peripheral blood by Sanger sequencing are consistent with segmental neurofibromatosis caused by a postzygotic mosaic NF1 variant.

## 5 Discussion

NF1 is an autosomal dominant neurocutaneous disorder caused by mutations in the *NF1* gene, with a global incidence of approximately 1 in 3,000 individuals (Rasmussen and Friedman, 2000). NF1 encodes neurofibromin, a 2818-amino acid protein that functions as a RAS GTPase-activating protein and acts as a tumor suppressor by negatively regulating the RAS/MAPK signaling pathway. Loss-of-function mutations in NF1 lead to hyperactivation of RAS signaling, dysregulated cell proliferation, and tumorigenesis (Upadhyaya et al., 1994; Viskochil, 2002). Among the spectrum of NF1-associated tumors, plexiform neurofibromas (PN) are benign but potentially debilitating, occurring in up to 50% of patients. PN often manifest in childhood, when tumor growth is most rapid, and may result in motor dysfunction, chronic pain, and severe disfigurement. While typical NF1 is characterized by widespread cutaneous and systemic manifestations, segmental neurofibromatosis—in which lesions are restricted to a single body region is rare and represents a unique clinical subset with distinct molecular characteristics (Anderson et al., 2022; Armstrong et al., 2023; Fisher et al., 2022).

In this study, we identified a novel intronic NF1 variant, c.7970+4_7970+7del, in the proband and demonstrated its pathogenic effect on pre-mRNA splicing using an *in vitro* minigene assay. This variant is located at the +4 to +7 positions of the donor splice site, a region that, although outside the canonical GT dinucleotide, contributes substantially to splice-site recognition and strength (Krawczak et al., 2007; Wimmer et al., 2007). Disruption of these conserved nucleotides may impair U1 snRNP binding and lead to aberrant splicing, including exon skipping or activation of cryptic splice sites. Consistent with this mechanism, the mutant minigene construct produced complete skipping of NF1 exon 54, whereas the wild-type construct showed normal splicing. These findings provide evidence that c.7970+4_7970+7del is a splice-altering NF1 variant. Exon 54 skipping is predicted to cause a frameshift and premature termination of neurofibromin translation, supporting a loss-of-function effect. This aberrant transcript may undergo nonsense mediated mRNA decay, and if translated, may generate a truncated neurofibromin product. Both mechanisms are consistent with NF1 loss of function. Neurofibromin acts as a negative regulator of RAS signaling through its GTPase-activating protein-related domain; therefore, NF1 loss of function can result in increased RAS-GTP activity and persistent activation of downstream RAS/RAF/MEK/ERK signaling. Although RAS/MAPK pathway activation was not directly assessed in this patient, the predicted consequence of this splice-altering variant is consistent with the established molecular mechanism of NF1-related tumorigenesis. In the context of segmental neurofibromatosis, a postzygotic NF1 loss-of-function variant may lead to localized neurofibromin deficiency in a subset of precursor cells, thereby contributing to regionally restricted neurofibroma formation.

The observed VAF of 36.02% in lesional tissue provides important clues regarding the developmental timing and biological behavior of the mutation. A VAF in this intermediate range is consistent with a somatic mutation arising during early to mid-embryogenesis, prior to the final differentiation of neural-crest-derived tissues. This mosaic distribution of mutant cells explains the localized pattern of neurofibroma formation while preserving normal phenotype elsewhere (Tinschert et al., 2000). Notably, the VAF is too high to be explained by subclonal expansion within a benign neurofibroma alone, supporting the interpretation of a developmental mosaic rather than an acquired tumor-specific event. From a clinical perspective, the mosaic status carries significant implications for reproductive risk. While the recurrence risk in offspring is substantially lower than the 50% risk observed in germline *NF1*, it is not negligible. The presence of a ~36% VAF raises the possibility of gonadal mosaicism, which has been documented in segmental NF1 and may account for familial transmission in rare cases. Genetic counseling should therefore emphasize that the risk for affected offspring, though low, is above that of the general population.

Splice-altering variants in *NF1* are increasingly recognized as important contributors to disease pathogenesis. Approximately 2–15% of reported NF1 pathogenic variants disrupt RNA splicing, often by generating cryptic splice donor or acceptor sites (Ars et al., 2000; Vandenbroucke et al., 2002; Wimmer et al., 2007). While canonical splice site variants are readily predicted, noncanonical intronic or exonic changes—including missense, nonsense, or synonymous variants—can also perturb splicing, highlighting the necessity of functional validation through RNA-based assays (Anna and Monika, 2018; Valero et al., 2011; Wimmer et al., 2007). In our study, the combination of *in vitro* minigene assay and in vitro minigene assays provided robust evidence that the c.7970+4_7970+7del variant is pathogenic, emphasizing the importance of integrating molecular and phenotypic data in NF1 variant interpretation. The rarity of segmental NF1, especially with isolated facial involvement, underscores the clinical significance of our findings. Segmental NF1 cases are often underdiagnosed due to the absence of typical systemic features such as café-au-lait macules, axillary freckling, or Lisch nodules. Our report provides direct molecular evidence linking a somatic splice-altering NF1 mutation to a localized neurofibroma phenotype, contributing to the understanding of genotype–phenotype correlations in this rare NF1 subset (Ejerskov et al., 2021; Tinschert et al., 2000).

Although mosaic NF1 is rare, the case contributes valuable insights into its molecular basis. Segmental manifestations likely result from early postzygotic *NF1* mutations that abolish neurofibromin activity in a subset of precursor cells. The degree and anatomical distribution of lesions depend on the affected cell lineages and timing of mutation. Our findings support this developmental model and illustrate how functional consequences at the molecular level translate into the observed circumscribed clinical presentation. This case also expands the known mutational spectrum of somatic *NF1* alterations. Few reports have functionally validated intronic splice-site variants in segmental neurofibromatosis, and even fewer have used minigene assays to elucidate their mechanistic effects. By providing both genomic and functional evidence, our study strengthens the genotype–phenotype relationship in this rare *NF1* subset.

Surgery remains the conventional treatment for symptomatic or disfiguring NF1-associated plexiform neurofibromas, but complete resection is often limited by the infiltrative nature of these tumors (Fisher et al., 2021). MEK inhibitors have recently provided a non-surgical therapeutic option for patients with symptomatic, inoperable NF1-associated plexiform neurofibromas (Gross et al., 2023; Weiss et al., 2021). Although our patient has not yet initiated systemic therapy because of financial constraints, the identification of a functionally validated NF1 loss-of-function variant provides a molecular rationale for considering targeted therapy during future management s (Packer et al., 2002).

In conclusion, we identified a novel somatic intronic NF1 splice-site variant, c.7970+4_7970+7del, in lesional tissue from a patient with segmental neurofibromatosis. Functional minigene analysis demonstrated complete exon 54 skipping, resulting in a predicted frameshift and premature termination. Although endogenous NF1 mRNA levels and neurofibromin protein expression were not directly assessed, the aberrant transcript may undergo nonsense-mediated mRNA decay and/or lead to NF1 loss of function. This case expands the functional evidence for splice-altering NF1 variants, emphasizes the importance of functional validation for splice-altering variants, and highlights the diagnostic and genetic counseling relevance of recognizing mosaic NF1 (Thomas et al., 2012).

## Data Availability

The data supporting the conclusions of this article will be made available by the corresponding authors upon reasonable request.

## Footnotes

1
https://rddc.tsinghua-gd.org/searchmiddle?to=SplitToolModel

2
https://rddc.tsinghua-gd.org/zh/tool/rna-splicer
