# Supplementary material for: Case Report: A somatic NF1 splice-altering variant identified in lesional tissue in peripheral blood-negative segmental facial neurofibromatosis
Source: Front Neurosci. 2026 Aug 12;20:1887523. doi: 10.3389/fnins.2026.1887523 (PMC13506871; doi:10.3389/fnins.2026.1887523)
Supplement: Supplementary file 1 [file Table_1.DOCX]

**Supplementary Table 1.** Summary of NF1 c.7970+4_7970+7del variant characteristics.

| **Parameter** | **Description** |
| --- | --- |
| Gene | NF1 |
| Transcript | NM_001042492.3 |
| Genomic position | NC_000017.11 |
| Variant | c.7970+4_7970+7del |
| Variant type | Intronic deletion (splice-site region) |
| Affected splice-site region | Donor splice site, +4 to +7 nucleotides |
| Population frequency | Not found in gnomAD, ExAC, 1000 Genomes |
| In silico splicing prediction | SpliceAI: donor-site loss; MaxEntScan: reduced score |
| Predicted RNA effect | Exon 54 skipping |
| Predicted protein effect | Frameshift → premature stop (p.Thr2625Ter) |
| Variant allele fraction (VAF) | 36.02% (206 WT reads : 116 MUT reads) |
| Mosaicism | Consistent with somatic mosaic NF1 |
| ACMG classification | Pathogenic (PVS1_Strong + PS3 + PM2) |
| Functional evidence | Minigene assay confirms exon 54 skipping |
